# Supplementary material for: Symptom improvement in adenomyosis patients after ultrasound guided microwave ablation or uterine artery embolization, A randomized controlled pilot study
Source: PLoS One. 2026 Mar 9;21(3):e0343949. doi: 10.1371/journal.pone.0343949 (PMC12970886; doi:10.1371/journal.pone.0343949)
Supplement: Attachment — Submitted filename: Response to reviewers PlosOne 251117.docx [file pone.0343949.s002.docx]

Dear Authors,

Thank you for submitting your manuscript entitled "Symptom improvement in adenomyosis patients after ultrasound-guided microwave ablation or uterine artery embolization: a randomized controlled pilot study."

Major statistical critiques:

Primary Endpoint and Multiple Comparisons:
The primary endpoint (Symptom Severity Score, SSS) showed no statistically significant improvement in the UAE group (p=0.067), indicating this is a negative study regarding the primary endpoint. It is important to clearly state this finding in your manuscript.

Thank you for this important comment, we have now clarified this both in the abstract (line 46 “There was no significant difference in primary outcome between the groups”) and in Conclusion (line 375 “no difference between the groups was seen regarding primary outcome”).

We have also tried to clarify the similar treatment effects by adding a new figure (replacing former figure 2), describing symptom alleviation after treatment in both groups, with no significant differences between the groups.

Moreover, several secondary endpoints reported as statistically significant may lose significance if appropriate adjustments for multiple comparisons are applied. The manuscript must explicitly acknowledge the negative primary endpoint and discuss the impact of multiple comparison corrections.

We found this suggestion very appropriate, and after discussion with our statistician, we have now added a Bonferroni correction regarding both Table 1 and Table 2. In Table 1, no significant differences between the two groups were seen after Bonferroni correction, hence the removal of the p-value column and instead an explanation in table legend.

In Table 2 one significant difference remained (hospitalization).

Regarding exploratory outcomes, the only remaining significant difference was “return to daily activities” which is explained in the text (Results, line 279-281).

We chose to remove Figure 2 entirely, since the two significant findings (postop pain day 2 and 3) did not remain significant after Bonferroni correction.

Endpoint Clarification and Table Presentation:
The manuscript currently lacks clear delineation of primary, secondary, and exploratory endpoints. Table 2 includes only primary and secondary endpoints; however, additional p-values for hospitalization duration and analgesic use are reported in both the abstract and results without classification. Please clearly define and organize these endpoints in the manuscript, preferably in a dedicated table distinguishing primary, secondary, and exploratory outcomes.

Thank you for this valuable comment; this was not appropriately described in our first draft. To address this, we have now arranged Table 2 as suggested, with clear headlines describing Primary and Secondary outcomes.

One of the secondary outcomes – Acceptability – do still need to be separately discussed, as it is calculated with a different statistical method, this is mentioned in the Results section (line 287-295).

The exploratory outcomes are now stated clearly in the Method section (line 205-206), in the Result section (line 279-282) and lastly in the Discussion section (line 317-321).

Power Analysis and Hypothesis Testing:
Given the absence of a formal power analysis (which you acknowledge), formal hypothesis testing is not appropriate for this pilot study. It is advisable to focus on estimation of treatment effects, effect sizes, and descriptive statistics rather than hypothesis-driven p-values. This approach will more appropriately reflect the exploratory nature of the study.

We totally agree with the reviewer regarding this important remark, and we have made several changes to address this valuable suggestion:

All p-values were removed from Table 1 (baseline characteristics) after performing Bonferroni correction.

In Table 2 (results of primary and secondary outcomes), p-values are presented as “n.s” when non-significant (only one significant difference remained after Bonferroni correction).

We have also removed all non-significant p-values from the text in the Result section, and discussed the risk of multiple comparisons in a limited patient material (Discussion, line 317-321).

Figure 2 is removed entirely, and has been replaced with a new figure, focusing on main symptom alleviation after treatment (in %), and the non-significant differences between the two groups are hopefully described clearly in this figure. We hope that this figure is descriptive regarding the clinical outcomes, and also demonstrating the lack of significant differences between the treatment groups.

Statistical Methodology and Baseline Imbalances:
The Mann-Whitney U-test used to compare groups does not adequately account for baseline imbalances, notably the significant baseline difference in CA-125 (p=0.03). A preferable statistical approach would be analysis of covariance (ANCOVA) or a mixed-effects model, which appropriately controls for baseline covariates and provides more accurate estimates of treatment effects.

With the aid of our statistician we have addressed this important remark by applying Bonferroni correction on all secondary and exploratory outcomes, resulting in only two remaining significant differences. According to our statistician, the material was not appropriate (too small) for other multivariate models such as ANOVA.

Confidence Interval Reporting:
The manuscript currently reports medians and p-values but lacks 95% confidence intervals for key outcomes. Including confidence intervals is essential for accurately interpreting the range and precision of the estimated treatment effects. Please include 95% confidence intervals for all key reported outcomes.

Thank you for this comment, we have now added CI 95% as suggested.

Addressing these statistical critiques will greatly enhance the robustness, clarity, and clinical interpretability of your manuscript.

Thank you for your attention to these important points and we look forward to receiving your resubmission.

Sincerely,

Plos One editorial office
