# Supplementary material for: Symptom improvement in adenomyosis patients after ultrasound guided microwave ablation or uterine artery embolization, A randomized controlled pilot study
Source: PLoS One. 2026 Mar 9;21(3):e0343949. doi: 10.1371/journal.pone.0343949 (PMC12970886; doi:10.1371/journal.pone.0343949)
Supplement: Attachment — Submitted filename: Response to Reviewers .docx [file pone.0343949.s003.docx]

**Response to Reviewers regarding PONE-D-25-59928**

Dear Reviewers and Editor,

Thank you for fruitful feedback. The manuscript has now been revised according to your suggestions, and we hope you can consider it for publication.

**Remark 1:** *The manuscript is formatted to PLOS ONE´s style requirements.*

**Remark 2,3 - added as suggested:**

*”The study was funded by The Stockholm County Council. HKK received the funding, the sponsors were not involved in anything connected to the study.”*

*"The funders had no role in study design, data collection and analysis, decision to publish, or preparation of the manuscript."*

*”According to the General Data Protection Regulation (GDPR) of the European Union (EU), no data containing personal information can be shared outside the EU unless patients have consented (which the patients in the current study have not). Data can therefore not be uploaded in a repository, nor disclosed within the manuscript or uploaded as supplementary information. Data can be shared within the EU after establishment of an agreement.”*

**Remark 4:** *title on the online submission form and title in the manuscript are identical*

**Remark 5:** *ethics statement is now only apparent in the Methods section, as suggested*

**Remark 6:** *non applicable*

**Remark 7:** *non applicable*

**Specific remarks from reviewer#2:**

Tables 1 and 2 now report unadjusted p-values and the Bonferroni correction have been deleted, as suggested by the reviewer.

The statistical test used for within-group pre–post comparisons and summary statistics (median with IQR and range) for post–pre differences within each treatment group have been clarified in the section of statistical analysis, as suggested by the reviewer:

*“To compare the difference between variables that were measured before and after treatment within each group we used Paired T test and descriptive statistics.” (line 217-219)*

Regarding postoperative outcomes, there was a significant difference between the groups in favor of MWA treatment: hospitalization was shorter (median 0 days compared to 3 days in the UAE group, p=0,004) and shorter time of postoperative pain medication (median 3 days compared to 10 days in the UAE group, p=0,029).
